# Volvulus du grêle sur lipome du mésentère

**DOI:** 10.11604/pamj.2017.27.76.12748

**Published:** 2017-06-01

**Authors:** Papa Alassane Mbaye, Aime Lakh Faye, Aloise Sagna, Ndeye Aby Ndoye, Ndeye Fatou Seck, Oumar Ndour, Gabriel Ngom

**Affiliations:** 1 Université Cheikh Anta Diop Service de Chirurgie Pédiatrique Hôpital d’Enfants Albert Royer, Dakar, Sénégal; 2 Université Cheikh Anta Diop Service de Chirurgie Pédiatrique Hôpital Aristide Ledantec , Dakar, Sénégal

**Keywords:** Lipome, mésentère, volvulus, enfant, Lipoma, mesentery, volvulus, child

## Abstract

Nous rapportons l'observation d'une fillette âgée de 7ans qui reçue dans un tableau de syndrome sub-occlusif avec une douleur abdominale aigue paroxystique siégeant au niveau de l'épigastre associée à des vomissements et un arrêt des matières. L'examen physique mettait en évidence une sensibilité à la palpation de l'épigastre. L'échographie abdominale a montré une formation tissulaire intra péritonéale mal limitée sans caractère vasculaire au doppler, exerçant un effet de masse sur les structures de voisinage ; les vaisseaux mésentériques étaient en position normale. A la tomodensitométrie cette masse correspondait à une formation lipomateuse bien limitée exerçant un effet de masse sur le caecum avec un volvulus du grêle. Le diagnostic de volvulus du grêle sur lipome mésentérique a été retenu. L'exploration chirurgicale confirmait ce diagnostic. Une détorsion grélique et une énucléation lipomateuse étaient réalisées. Les suites opératoires étaient simples après un recul de 6 mois. L'examen anatomopathologique confirmait la nature lipomateuse de la masse.

## Abstract (en)

We report the case of a 7-year old girl presenting with sub-occlusive syndrome associated with acute paroxysmal abdominal pain at the level of the upper abdomen, vomiting and no evacuation of faeces. Physical examination showed discomfort with palpation of the upper abdomen. Abdominal ultrasound showed poorly limited intraperitoneal tissue formation without vascular features on doppler, exercising a mass effect on the neighborhood structures; mesenteric vessels were in their normal position. This mass on CT scan scorresponded to a well limited lipomatous formation exercising a mass effect on the caecum associated with volvulus of the small intestine. The diagnosis of volvulus of the small intestine caused by mesenteric lipoma was retained. Surgical exploration confirmed this diagnosis. The patient underwent unrolling of the small intestine and lipoma enucleation. The postoperative course was uneventful after a follow-up of 6 months. Anatomo-pathological examination confirmed the lipomateuse nature of the mass.

## Introduction

Le lipome est une tumeur bénigne constituée de tissu adipeux qui n'entraîne généralement pas de complications [1]. Sa localisation mésentérique est rare. Le volvulus du grêle sur lipome mésentérique est exceptionnel [2]. Nous rapportons cette affection à propos d'un cas afin de mettre en évidence ses aspects épidémiologiques, diagnostiques et thérapeutiques.

## Patient et observation

**Figure 1:**
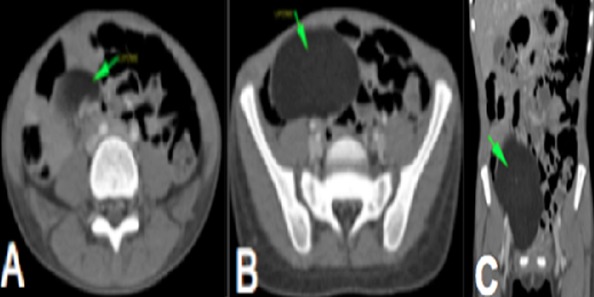
(A, B) Coupes axiales de scanner abdominal avec injection de produit de contraste montrant une masse de densité graisseuse homogène non rehaussée au niveau du flanc et de la fosse iliaque droite comprimant le cœcum en rapport avec un lipome; C) reconstruction coronale de scanner abdominal injecté montrant la masse graisseuse et ses limites

**Figure 2:**
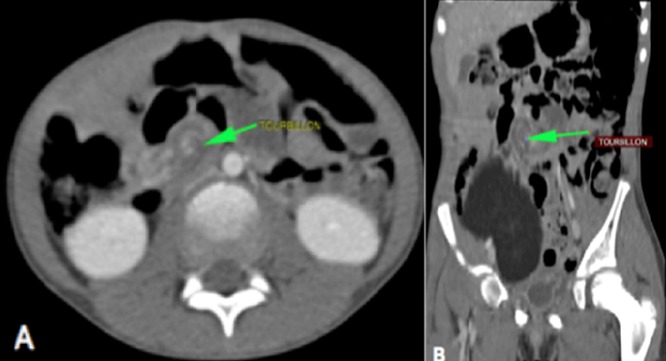
A) Coupe axiale; B) reconstruction coronale de scanner abdominal injecté montrant le volvulus du grele (flèche verte) juste au-dessus de la masse lipomateuse en rapport avec le volvulus du mésentère

**Figure 3:**
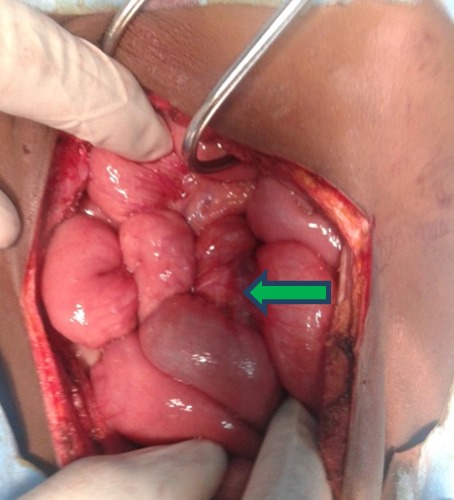
Image peropératoire montrant le volvulus du grêle

**Figure 4:**
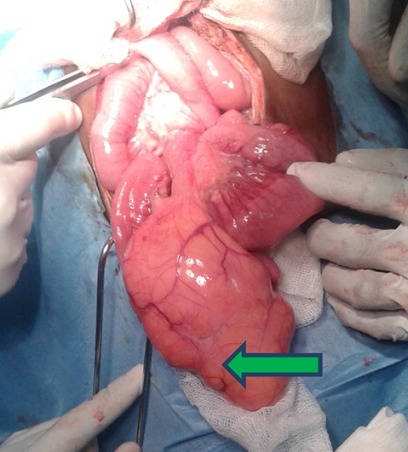
Volumineux lipome du mésentère après détorsion manuelle du grêle dont une partie était comprise dans le volvulus

Notre observation concerne une fillette de 7 ans qui a été adressée au service de chirurgie pédiatrique de l'hôpital Aristide Ledantec pour la prise en charge d'un syndrome sub occlusif évoluant depuis 6 jours. Le début était marqué par des douleurs abdominales de localisation épigastrique évoluant de manière paroxystique avec des périodes d'accalmie. Elle présentait des vomissements alimentaires et un arrêt des matières. Elle avait un bon état général et des muqueuses conjonctivales colorées. A l'examen physique l'abdomen était météorisé, sensible au niveau de l'épigastre. Le toucher rectal était sans particularité. La numération formule sanguine, l'ionogramme sanguin et le bilan de l'hémostase étaient normaux. L'échographie a montré une masse tissulaire mal limitée présentant des contours polylobés sans caractère vasculaire exerçant un effet de masse sur les structures de voisinage. Les vaisseaux mésentériques étaient normalement situés. La tomodensitométrie concluait à un volvulus du grêle au voisinage d'une masse d'allure lipomateuse en rapport avec le mésentère (Figure 1, Figure 2). Nous avons réalisé une laparotomie exploratrice par une incision transversale sus ombilicale. L'exploration chirurgicale a retrouvé un volvulus du grêle à deux tours de spires dans le sens antihoraire à environ 70 cm de l'angle iléo-caecal (Figure 3), sans signe de souffrance intestinale. Elle mettait aussi en évidence une masse lipomateuse sur le mésentère dont une partie était intégrée dans le volvulus (Figure 4). Une détorsion manuelle du volvulus a été réalisée associée à une exérèse complète de la masse par ouverture du feuillet antérieur du mésentère. Les suites opératoires étaient simples après un recul de 6 mois .L'examen anatomopathologique concluait à une formation tissulaire lipomateuse.

## Discussion

Le volvulus du grêle chez l'enfant est le plus souvent secondaire à une malrotation intestinale [3]. Il est rarement secondaire à un lipome mésentérique. Chez l'enfant, les lipomes sont superficiels au niveau du tronc. Les lipomes profonds peuvent intéresser le thorax, le médiastin et rarement le mésentère intestinal [4]. Le diagnostic positif de volvulus sur lipome du mésentère se fait souvent au moment de la laparotomie, car la symptomatologie clinique est très polymorphe et non spécifique [3–5]. Il peut être asymptomatique, de découverte fortuite ou se manifester par une masse abdominale avec des signes digestifs faisant craindre une complication majeure tel qu'un volvulus [6]. C'est le cas de notre patiente avec un syndrome sub-occlusif pour lequel l'imagerie a permis la confirmation diagnostique. Les explorations radiologiques actuelles, notamment l'échographie et la tomodensitométrie, constituent un élément important du diagnostic. Cependant, les autres types de tumeurs du tissu graisseux tel que le liposarcome ne peuvent être formellement écartés avant d'avoir une preuve histologique [4,6]. L'échographie couplée au doppler est d'un apport important permettant de mettre en évidence le volvulus du grêle et ses tours de spires [7]. De plus elle permet d'évoquer une souffrance des anses digestives devant l'épaississement de la paroi des anses au niveau du volvulus [7,8]. Enfin cet examen est facile à réaliser, non invasif et non douloureux [1]. Chez notre patient l'échographie a permis de mettre en évidence une masse tissulaire avasculaire dont la nature n'a pas été précisée avec effet de masse sur les structures de voisinage.

L'étude faite par Sadra [9 ,10] a montré que l'échographie n'est pas toujours performante, car les limites de la tumeur sont imprécises et la localisation exacte par rapport au péritoine est difficile à préciser avec exactitude. De même l'échogénicité est variable d'un lipome à un autre. C'est le cas pour notre patient. Couplée à l'échographie, la tomodensitométrie permet d'appuyer le diagnostic du lipome du mésentère en permettant d'étudier la densité de la tumeur, sa nature graisseuse, sa localisation exacte, sa taille et son étendue [1,11]. En plus, elle détermine avec exactitude les rapports avec les organes de voisinage. Elle a l'avantage aussi d'éliminer certains diagnostics différentiels, notamment le tératome par l'absence de calcifications. Le lipome du mésentère se présente sur le plan scannographique comme une masse intraperitionéale, encapsulée, de densité graisseuse, contenant des travées fines non rehaussées par le produit du contraste, sans rapport avec les organes de voisinage, tout en précisant sa taille [4,12]. Chez notre malade la tomodensitométrie nous a permis de retenir le diagnostic en mettant en évidence un volvulus du grêle et de confirmer la nature lipomateuse de la masse. Le traitement chirurgical demeure actuellement le traitement de choix du volvulus sur lipome du mésentère [7]. Deux méthodes sont décrites: la laparotomie classique et la laparoscopie. Ce traitement constitue le temps le plus important aussi bien pour confirmer le diagnostic par l'examen anatomopathologique de la pièce d'exérèse, que pour réaliser la détorsion intestinale [7]. La laparoscopie constitue actuellement l'examen de choix aussi bien pour le diagnostic que pour le traitement du lipome du mésentère, en permettant d'une part, de préjuger de la nature de la tumeur et de son aspect macroscopique, et d'autre part, de faire une exérèse complète de la tumeur [7]. Le diagnostic de volvulus sur lipome du mésentère se fait le plus souvent au moment la laparotomie. Chez l'enfant l'incision transversale est la plus utilisée [4, 9,13] comme ce fut le cas pour notre patient. La détorsion intestinale et l'exérèse complète constituent les temps forts de la prise en charge chirurgicale en l'absence de nécrose intestinale. Cette exérèse permet d'éviter la dégénérescence sarcomateuse et les autres complications du volvulus [12]. Chez notre malade nous avons réalisé une laparotomie transversale au niveau du pli abdominal supérieur (absence de colonne de coelioscopie), une détorsion grélique suivie de l'exérèse de la masse. L'étude anatomopathologique permet de confirmer le diagnostic histologique.

## Conclusion

Le lipome du mésentère est une affection bénigne rare chez l'enfant, Il touche surtout le mésentère de l'intestin grêle. Cliniquement, il est soit asymptomatique ou révélé à l'occasion d'une symptomatologie non spécifique ou d'une complication telle que le volvulus du grêle. L'échographie n'est pas toujours fiable pour poser le diagnostic d'où la nécessité de réaliser une tomodensitométrie. Son traitement reste chirurgical.

## Conflits d’intérêts

Les auteurs ne déclarent aucun conflit d'intérêt.
